# Organic versus Conventional Raw Cow Milk as Material for Processing

**DOI:** 10.3390/ani11102760

**Published:** 2021-09-22

**Authors:** Aneta Brodziak, Joanna Wajs, Maria Zuba-Ciszewska, Jolanta Król, Magdalena Stobiecka, Anna Jańczuk

**Affiliations:** 1 Institute of Quality Assessment and Processing of Animal Products, Faculty of Animal Sciences and Bioeconomy, University of Life Sciences in Lublin, Akademicka 13, 20-950 Lublin, Poland; aneta.brodziak@up.lublin.pl (A.B.); jolanta.krol@up.lublin.pl (J.K.); magdalena.stobiecka@student.up.edu.pl (M.S.); annajanczuk@student.up.edu.pl (A.J.); 2 Institute of Economics and Finance, Faculty of Social Sciences, The John Paul II Catholic University of Lublin, Racławickie 14, 20-950 Lublin, Poland; maria.zuba@kul.pl

**Keywords:** milk, production system, organic dairy products, yoghurt, cheese, bioactive compounds, mycotoxins, technological parameters

## Abstract

**Simple Summary:**

Milk, alongside meat, is one of the basic animal materials of importance in food processing. Most of the world’s milk production is carried out in an intensive system focused on high productivity at the expense of quality. This system is dominant in highly developed countries, while milk production in developing and poorer countries is still carried out in a traditional manner, using silage. The popularity of certified organic milk has been increasing in European countries since the 1990s. The aim of the article was to compare the quality of raw milk from three production systems, intensive, traditional, and organic, as material for processing. Research assessing the quality of organic milk and dairy products is much less extensive than for conventionally produced milk (intensive and traditional). The available reports indicate that raw milk from organic farms is more valuable, particularly in terms of the content of health-promoting compounds, including vitamins, fatty acids, whey proteins, and minerals. This stems from the fact that animals in organic farming are kept in the pasture. However, the hygienic quality of the raw milk raises some concerns, and organic milk producers should be supported in this regard, e.g., through consultancy and training. Importantly, milk production in traditional and organic systems is in line with the concept of the European Green Deal.

**Abstract:**

Milk, as one of the basic raw materials of animal origin, must be of adequate hygienic and physicochemical quality for processing. The aim of the article was to compare the quality of raw milk from three production systems, intensive, traditional (together referred to as conventional), and organic, as material for processing, as well as the quality of products made from it. Particular attention was focused on hygienic quality (somatic cell count and total bacterial count), physical characteristics (acidity), basic nutritional value (content of dry matter, total protein, casein, fat, and lactose), content of health-promoting substances (whey proteins, fatty acids, vitamins, and minerals), and technological parameters (rennet clotting time, heat stability, and protein-to-fat ratio). Research assessing the quality of organic milk and dairy products is significantly less extensive (if available at all) than for milk from conventional production (intensive and traditional). The available reports indicate that raw milk from organic farms is more valuable, especially in terms of the content of health-promoting compounds, including vitamins, fatty acids, whey proteins, and minerals. This applies to organic dairy products as well, mainly cheese and yoghurt. This is explained by the fact that organic farming requires that animals are kept in the pasture. However, the hygienic quality of the raw milk, and often the products as well, raises some concerns; for this reason, organic milk producers should be supported in this regard, e.g., through consultancy and training in Good Hygienic Practices. Importantly, milk production in the traditional and organic systems is in line with the concept of the European Green Deal.

## 1. Introduction

Milk, alongside meat, is one of the basic animal materials of importance in food processing. Dairy production around the world, including Poland, is dominated by cow milk. This is due to the higher productivity of cows compared to other dairy species. Moreover, it is the most universal raw milk for processing due to its specific content and proportions of proteins, fat, and mineral compounds [1,2,3,4,5,6,7].

Global production of cow milk in 2019 amounted to almost 716 million tonnes and was 36 times higher than production of goat’s milk and nearly 68 times higher than production of sheep’s milk. Since 2000, global production of cow milk has increased by over 46%. Europe remains the largest producer of this raw material. However, its percentage in world production decreased by almost 11 p.p. to 31.5%, mainly due to a small increase in production (8.6%) compared to other parts of the world, such as Asia (133%). Due to the 12.5% increase in the production of cow milk in the EU28, milk production in these countries has increased its share of production in Europe (by 2.5 p.p. to 74.5%), while the role of the EU globally has decreased (by 7 p.p. to 23.5%) [8,9].

**Figure 1:**
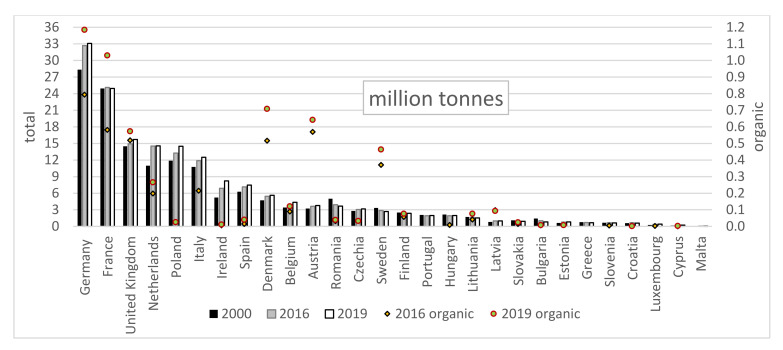
Production of cow milk, including organic milk, in the EU28 * (own work based on [8,11]). * data on organic milk not available for Portugal, Greece and Malta.

The largest producers of cow milk in the EU as of 2019 were Germany, France, the United Kingdom, the Netherlands, Poland, and Italy (Figure 1), with a total share of 68.5% (115.29 million tonnes), which has remained practically unchanged since 2000. The volume of milk production increased in countries with high (Germany, The United Kingdom, The Netherlands, Poland, and Italy), medium (Belgium, Denmark, Ireland, Spain, Austria, Czech Republic), and low production (Estonia, Cyprus, Latvia, Luxembourg). Production of organic milk in the EU grew dynamically in recent years (126% since 2007) to 5.52 million tonnes in 2019 and was expected to exceed 6 million tonnes from 2020 [10]. Currently, nearly 3.3% of milk produced is organic, and this proportion is growing steadily (it has doubled since 2007). The largest producers of organic milk in the EU in 2029 were Germany (1.19 million tonnes in 2019), France (1.03 million tonnes), Denmark (0.71 million tonnes), Austria (0.64 million tonnes), The United Kingdom (0.57 million tonnes), and Sweden (0.46 million tonnes). This accounted for 83.4% of organic production in the EU. Organic milk production has been growing for several years, although total milk production in some of the countries (Sweden and France) is decreasing. Organic production in the EU is also becoming more concentrated, as the share of the six largest producers of organic raw milk is increasing (1.4% since 2016). The percentage of organic milk in total milk production in the EU is highest in countries with average or small milk production volume, i.e., Sweden (17.2%), Austria (17.0%), Denmark (12.6%), and Latvia (9.6%). In other countries with organic production, its proportion does not exceed 5%. A surprising phenomenon is the lack of growth of organic milk production in countries with high (Poland) or medium (Ireland, Spain) total milk production [8,11].

**Figure 2:**
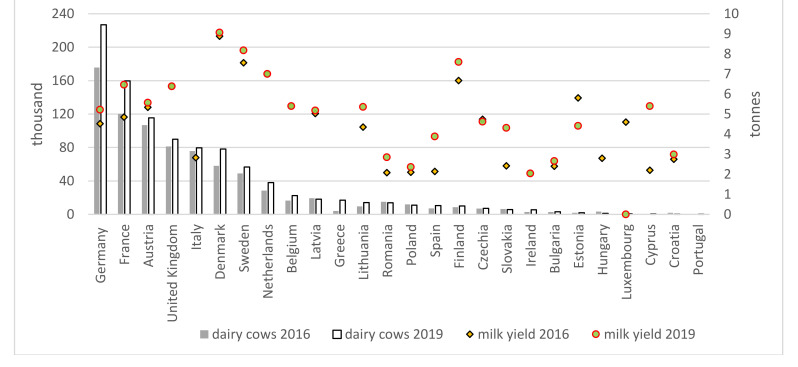
Number of cows on organic farms and their milk yield in the EU28 * (own work based on [8]). * data on milk yield not available for Greece and Portugal. * data not available for Malta and Greece.

The high production volume of organic milk in some countries is mainly due to their large dairy cow populations (Germany, France, Austria, and The United Kingdom) and high milk yield in the case of Denmark and Sweden (over 8000 kg in 2019). In these countries, both the number of cows kept on organic farms and their productivity are increasing (Figure 2). In Italy, despite the large cow population, milk production is low due to poor milk yield. In Poland, the milk yield of cows is improving steadily (34.5% to 2362.8 kg since 2012), but the number of cows continues to decline (by 45% to less than 11,000 in 2019), which has reduced milk production (by 26% to almost 26,000 kg) [8].

**Figure 3:**
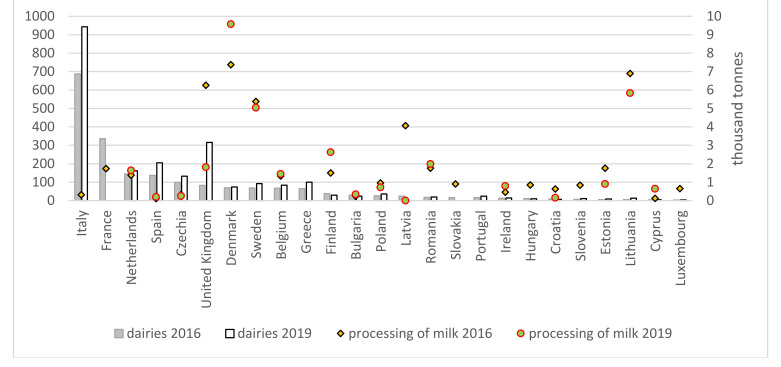
Number of organic dairies and average volume of milk processed in the EU28 * (own work based on [8]). * data on processing of milk not available for Greece and Portugal.

The technical efficiency of organic milk processing is highest in Denmark, Lithuania and Sweden (over 5000 tonnes of milk in 2019; Figure 3). These are countries with high milk production (Denmark and Sweden) or low production and few dairies (Lithuania). The most certified dairies are located in Italy, France, the Netherlands, Spain, the Czech Republic, and the United Kingdom. The United Kingdom is an interesting example of a lack of coordination between the rate of milk production and its processing, as the performance of dairy plants decreased by 71% within a few years due to the rapid increase in the number of dairies (from 83 to 316) accompanied by slower growth of organic milk production (by 10%). In Poland, the number of certified dairies has increased by almost 12% to 36 since 2012, which, given the decrease in the volume of raw milk produced (26%), resulted in a significant (65%) reduction in the technical efficiency of dairies to 721 tonnes [8].

The aim of the article was, therefore, to compare the quality of raw milk from three production systems, i.e., intensive, traditional, and organic, as material for processing. Research assessing the quality of organic milk and dairy products is much less extensive than for conventionally produced milk (intensive and traditional). Milk and dairy products from organic production are not fully recognized as a valuable source of nutrients, including health-promoting compounds which the authors decided to verify.

## 2. Requirements for Processing Raw Milk

For processors, what is most important is the quality of the raw milk, which is verified when the milk is received by the plant. Pursuant to Commission Regulation (EC) No 1662/2006 of 6 November 2006 relating to the hygiene of food of animal origin [12], the total bacterial count (TBC) and somatic cell count (SCC) in raw cow milk may not exceed 100,000 cfu/mL and 400,000 somatic cells/mL, respectively. Total bacterial count (TBC) has become one of the criteria adopted for grading milk around the world [13,14]. High-quality raw milk has a low TBC [15,16,17]. In the United States, the Pasteurized Milk Ordinance requires that bacterial and somatic cell counts of Grade A raw milk do not exceed 100,000 standard plate count (SPC) and 750,000 SCC/mL, respectively [18]. Raw milk must also meet other quality standards. It should be free of drug residues, added water, sediment, contaminants, and other abnormalities. The overall condition and cleanliness of the dairy farm, determined in routine inspections, are considered as well. In China, the national standard requires raw milk to have a TBC of <2 × 10^6^ cfu/mL [19]. Although regulatory requirements have been instrumental in ensuring the quality of raw milk, most segments of the dairy industry feel that more stringent standards should be maintained. Burke et al. [20] reports that in some US states an SPC of less than 10,000 cfu/mL is required for unpasteurized milk for direct consumption, which is regulated by state law [21]. In England and Wales, the SPC value must be lower than 20,000 cfu/mL in unpasteurized milk for direct consumption, while in Germany the limit for certified raw milk is 50,000 cfu/mL [21].

European law also regulates the temperature of milk. Directly after milking, milk must be cooled immediately to a temperature no higher than 8 °C in the case of daily collection or no more than 6 °C if it is not collected daily. Cooling conditions must also be maintained during transport; milk temperature must not exceed 10 °C on delivery to the plant [12]. Therefore, food business operators must implement procedures to ensure that raw milk meets these criteria. Additionally, there are specific national regulations in effect. In Poland, milk for processing must have an appropriate acidity, indicating its freshness, i.e., pH in the range of 6.6–6.8 and titratable acidity of 6.0–7.5 °SH [22]. The use of high-quality raw milk is a key factor determining the quality of dairy products. It is important to manufacturers because it ensures the desired taste and texture of the products as well as the efficiency of the physical processes the milk undergoes and its fermentation. This is especially important in cheese production [23]. Table 1 presents the characteristics of milk preferred for cheese-making.

Table 2 lists parameters for milk intended for the production of fermented milk beverages.

The quality of raw milk, however, is determined by many genetic (species, breed, variety) and environmental factors (feeding system, production season, welfare, climate zone) [1,3,26,27,28]. It is generally believed that more than 50% of the variability in the content of nutrients is determined by genetic factors and about 40% by environmental factors. The productivity of cows and the quality of milk are mainly determined by their diet, which is closely linked to the production system, and this in turn is largely associated with the production season [2,25,29].

## 3. Milk Production System

Most of the world’s milk production is carried out in a conventional, usually intensive system, focused on high productivity. This system is dominant in highly developed countries, while milk production in developing and poorer countries is still carried out in a traditional (extensive) manner. The popularity of organic milk, whose production is certified, has been increasing in European countries since the 1990s.

### 3.1. Organic System

Organic milk production may only be conducted on certified farms, supervised by a certification body. It must strictly comply with Council Regulation (EC) No 834/2007 [30] (detailed in Commission Regulation (EC) No 889/2008 [31]). The regulation remains in effect until the end of 2021, and from 1 January 2022 it will be replaced by Regulation (EU) 2018/848 of the European Parliament and of the Council of 30 May 2018 on organic production and labelling of organic products and repealing Council Regulation (EC) No 834/2007 [32]. Organic animal production combines the preservation of a high level of biodiversity, protection of natural resources, and high animal welfare standards. The specific rules in force on organic farms are conducive to the production of high-quality material. These include prioritization of native cattle breeds, animal nutrition based on on-farm feed from organically fertilized crops, prohibition of industrial concentrates, complete feeds, feed produced with GMOs, growth stimulants, synthetic amino acids and antibiotics, permanent access to outdoor areas, and stocking density adapted to the buildings and grassland areas [2,31,32]. According to the principles of organic production, animal feeding on organic farms is not aimed at intensive exploitation of cows, but at sustaining the balance of feed resources and utilizing them completely. Animals must be fed organic feed composed of ingredients obtained from organic agricultural production, but natural non-agricultural substances are also allowed. At least 60% of the feed must come from the same farm. Moreover, at least 60% of the dry matter in the feed ration should be roughage, green fodder, dried fodder, or silage. Pastures should be maximized accordingly to their availability in different seasons of the year [31,32,33]. The differences in non-conventional and organic feeding result mainly from the diversity of available vegetation in pastures, mainly in the spring and early summer. In summer, cows have ad libitum access to pasture vegetation, consisting mainly of low grasses (50%), tall grasses (30%), and legumes (10–20%). It should be emphasized that the pasture feeding period on organic farms often exceeds 180 days, while on traditional farms it usually lasts no longer than 140 days [34]. Organic pastures are distinguished by high sward biodiversity (numerous species of grasses, legumes, and herbs), which directly translates into the nutritional value and quality of fodder. This fodder is a source of numerous bioactive substances that enter the milk [2]. In the autumn and winter, cattle must be fed roughage, which includes silage based on mixtures of cereals and legumes or haylage. Beets or potatoes are added to the diet of cattle only in winter [35,36].

### 3.2. Conventional Systems

#### 3.2.1. Intensive System

One of the most popular feeding systems for high-yield cows is a total mixed ration (TMR) administered using a feed truck. TMR is a mixture of roughage and concentrate feeds supplemented with vitamin and minerals. The key feature of this system is the stability of the food ration, as frequent modifications may affect digestion or the occurrence of metabolic diseases in animals [37]. This system is used in high-yield cows that require a complete diet satisfying the demand for all necessary nutrients, deficiencies of which may negatively affect the quality and quantity of the product [38]. The basic ingredients of concentrate feed include maize silage, grass silage, legume silage, alfalfa hay, barley, or beet molasses/pulp [2,3,39,40]. The TMR system requires the formation of several feeding groups or the use of one averaged food ration. However, the use of an averaged ration does not allow for full utilization of the capacity of the most productive cows, while at the same time costly concentrate feed is given to cows with lower production potential. An alternative is PMR (partial mixed ration), a system, of partially complete feed rations. The PMR system combines the advantages of TMR and the precision of feeding stations in the rationing of concentrate feed and vitamin and mineral supplements. Moreover, PMR does not require separate feeding groups, because all cows receive one basic TMR ration composed for a specific average milk yield, and only individual cows exceeding this yield are rewarded with concentrated feed and additives from the feeding station. This system reduces the consumption of concentrate feed and is better adjusted to the energy and protein requirements of individual cows, e.g., those in the first stage of lactation [37,39,41]. An increasing number of dairy cow farms have introduced intensive milk production systems (TMR or PMR) in the last 20 years. This is linked to the construction of new barns or modernization of old tie-stall barns in favour of free stalls, in which a uniform, complete-ration feeding system is used throughout the year [42].

#### 3.2.2. Traditional System

The intensive milk production system is replacing the traditional system, which until recently was the most common, although the latter will continue to dominate for a long time in some parts of the world (especially in developing countries) [43,44,45,46]. On traditional farms, the cows are kept in tie-stall barns with or without litter. The diet is based mainly on roughage, usually administered ad libitum (without specialized equipment and calculation of feed rations). Grassland, both permanent and temporary, is an important source of fodder from spring to autumn in the traditional production system and is also the least expensive. The ration is not uniform in this feeding system. It is usually difficult to balance, and for this reason high milk yields are difficult to achieve. However, as a more natural system for cows, it is conducive to their welfare. The traditional and intensive systems collectively are referred to as the conventional milk production system. However, at the turn of the 21st century, their alternative, the organic system, has gained in popularity [47,48,49].

## 4. Milk Production System and the Quantity and Quality of Raw Milk and Manufactured Dairy Products

Research by many authors [2,26,27,28,50,51,52,53,54] indicates that the specific environmental conditions (mainly feed type and quality) on a farm focused on milk production affect the quality of raw milk, and consequently, dairy products.

### 4.1. Milk Yield

The key factor determining the profitability of dairy cow farms is their productivity. The influence of the feeding system on cows’ milk yield has been demonstrated by many authors [2,27,28,53,54,55,56,57,58,59]. In a comparison of organic and conventional systems by Bilik and Łopuszańska-Rusek [60], organic feeding of Red-and-White × Holstein-Friesian Red cows kept in natural conditions and having a lactation yield of about 7000 kg milk resulted in milk yield about 10% lower than that of cows fed conventionally (TMR) (6666.7 kg vs. 7353.4 kg). Wójcik-Saganek [57] studied Simmental cows fed in an intensive system (conventional farms) and traditionally (organic farms). The cows on the conventional farms also produced significantly (*p* ≤ 0.01) more milk (by 4.6 kg/day) than the cows on organic farms (20.9 vs. 16.3 kg/day). Similar trends were reported by Kuczyńska [51], who obtained significantly (*p* ≤ 0.01) higher productivity (by 10.32 kg/day) in Holstein-Friesian cows fed in the TMR system (25.48 kg/day) than in those fed according to principles of organic farming. The higher milk yield obtained in the intensive system was most likely a consequence of the better-balanced feed rations compared to organic farms, where the diets are not balanced at all. Król et al. [61] also demonstrated that the organic and traditional milk production systems did not fully satisfy the cows’ nutrient requirements. This was reflected statistically (*p* ≤ 0.01) by approximately 20% lower productivity of cows kept on organic and traditional farms (16.1 kg and 17.4 kg, respectively) compared to conventional farms (PMR) (22.3 kg). A study by Rosati and Aumaitre [58] also showed differences (approximately 10%) in the quantity of milk obtained, in favour of intensive systems vs organic herds. According to the authors, this was due to limitations on the use of certain ingredients in the cows’ diet and to the lower intensity of pasture fertilization on organic farms. Based on a meta-analysis, Średnicka-Tober et al. [62] showed that milk yields per cow were on average 20% lower in organic systems compared with conventional farms. Nauta et al. [59] carried out a comparative assessment of Dutch organic farms keeping Holstein cows, distinguishing between farms with a long tradition of organic certification and those being converted to organic farms, as well as conventional farms as a reference group. They observed that milk production was lower on long-standing organic farms than on conventional and converted organic farms. Interestingly, the milk production level on pre-organic farms, i.e., before their conversion, was already lower than on the conventional farms. Statistical analysis in this study showed a highly significant decrease in milk yield due to conversion.

### 4.2. Hygienic Quality

#### 4.2.1. Somatic Cell Count (SCC)

Somatic cell count (SCC) is a diagnostic index of the health of the mammary gland. A value above 400,000 cells in 1 mL of milk indicates inflammation of the gland. Inflammation negatively affects the productivity of cows, as well as the nutritional value of milk and its suitability for processing [63]. Wójcik-Saganek [57] reported that milk from Simmental cows fed in the TMR system had a significantly (*p* ≤ 0.01) lower SCC (231,000 cells/mL) than organic milk from the same breed (330,000 cells/mL). In both cases, however, the values were in compliance with EU requirements. Kuczyńska et al. [4] also showed a significantly (*p* ≤ 0.05) higher SCC in organic milk (206,000 cells/mL) compared to conventional milk (119,000 cells/mL). Nauta et al. [59] found that somatic cell counts were higher on long-standing organic farms than on conventional farms and converted organic farms. Other studies, however, have shown a higher somatic cell count in milk samples from conventional farms [51,64,65], while Haskell et al. [66] showed no significant effect of the milk production system on SCC. Therefore, it is difficult to conclusively determine a relationship between SCC and the production system. However, organic milk production is difficult, and the cows experience more frequent inflammations of the mammary gland. Therefore, it is recommended that the animals kept on these farms should be of local native breeds, which are more resistant to disease, including mastitis. Simmental [2], the second most popular dairy cattle breed in the world, is an example of such a local breed [9]. Simmental cows utilize roughage well, and thus do not require large amounts of concentrate fodder and intensive feeding. For this reason they are predisposed to the conditions of low-input and organic farms, including those situated in mountainous areas, where the diet is based on grazing on pastures and meadows in summer and on hay and haylage in winter. Moreover, they are highly tolerant of difficult environmental conditions in the foothills and mountains [2,67].

Dairy producers must take care to ensure adequate udder hygiene, as it directly affects the quality of raw milk, whose somatic cell count is verified at the dairy plant [12,68]. According to Moradi et al. [27], the actual effects of SCC on cheese making properties are not clear. Multiple concurrent factors may influence the final quality of these products. Milk SCC is an indicator of the presence of many compounds that may adversely affect milk and dairy products, including cheese. Somatic cells may increase the proteolytic and lipolytic activity of cheese, which increase the development of biogenic amines and off-flavours. According to Summer et al. [28], a high somatic cell count in milk is associated with a reduction in Parmigiano-Reggiano cheese yield, due to a decrease in both milk casein and recovery of milk fat in the cheese. Bobbo et al. [69] reported associations between pathogen-specific cases of subclinical mastitis and several milk composition traits (casein-to-protein ratio and lactose content) and cheese-making parameters (clotting ability).

#### 4.2.2. Microbiological Quality

The total bacterial count (TBC) is the second important parameter of milk hygiene. The number and types of microorganisms in milk immediately after milking are affected by milking hygiene (cleanliness of milking equipment and access to water) [70,71,72,73]. Total bacterial count is a good indicator for monitoring sanitary conditions during the production and handling of dairy products.

Luukkonen et al. [74] compared the chemical composition and hygienic quality of organic and conventional milk from 126 Finnish farms. Organic milk had a lower total bacterial count than conventional milk and a similar or higher somatic cell count. However, the authors noted that although the differences between organic and conventional milk were rather small, they were of economic significance in large-scale cheese manufacturing. Kapturowska et al. [75], in an assessment of the quality of raw milk in relation to the quality of ensiled roughage on selected organic farms, indicated a possible relationship between the quality of ensiled roughage and selected quality parameters of raw milk. In the milk from farms where cows were fed with experimental silage of high quality, including microbiological quality, no contamination or significantly less was detected, including microbiological contamination.

Good hygienic quality of milk and milk products is important for consumer health. There are several reports in the literature on the microbiological quality of organic dairy products. Berthold and Stachura [76] determined the microbiological quality of various types of cheese from organic farms (52 samples). The total bacteria count was 10^5^–10^10^ in curd cheeses, 10^7^–10^11^ in ripened cheeses, and 10^8^–10^11^ cfu/g in acid rennet cheeses, while the count of yeasts and moulds was 10^2^–10^5^, 10^1^–10^6^, and 10^1^–10^7^ cfu/g, respectively. According to the authors, the results showed significant flaws in the production and storage of cheeses made from organic raw milk. Psychrotrophic bacteria, a group of microorganisms active during refrigerated storage of products, were detected in 100%, 94%, and 74% of the samples of curd cheese, acid rennet cheese, and ripened rennet cheeses, respectively. Therefore, they recommended that producers of curd cheeses should lower the storage temperature of these cheeses to below 4 °C, which would minimize microbial growth and ensure the high quality of the cheese. In another Polish study by Kukułowicz [72] concerning pasteurized milk (2%) and dairy products (natural yoghurt, cream, cottage cheese, and ripened rennet cheese), whose main ingredient was pasteurized cow milk (58 products in total), *Salmonella* spp. bacteria were not found in any of the samples tested. *Escherichia coli* was not detected in conventional dairy products, while it was found in 7% of organic cheese samples. That percentage of samples of curd cheeses contaminated with *Escherichia coli* was significantly lower than that reported in the study by Berthold and Stachura [76]; however, as in that study, curds from organic production proved to be more contaminated with these microorganisms. A higher level of coliform bacteria, which adversely affects the microbiological quality of the product, could indicate a lower hygienic standard on organic farms [71,77]. Kouřimská et al. [71] found no differences between products in terms of the number of coliforms in milk from organic and conventional farming. The presence of enterococci, filamentous fungi, and yeasts in the products may also be indicative of production hygiene [72,76]. Dairy products studied by Kukułowicz [72] (cream, cottage cheese, and ripened rennet cheese) contained *Enterococcus* sp. Faecal streptococci are microorganisms commonly found in dairy products due to their ability to survive in unfavourable conditions [78]. Enterococci were found in over 33% of the tested creams and almost 67% of curd cheeses. The results were lower than in the findings of Bis and Mędrela-Kuder [79], who detected 92% and 75% contaminated samples of these products, respectively. *Staphylococcus aureus* can be found in dairy products due to the use of unsanitary practices, e.g., as a result of contamination from equipment, contact surfaces, floors, or packaging materials [80]. The average number of staphylococci obtained for curd cheeses from organic and conventional production was higher than the permissible limits specified in Commission Regulation (EC) No 2073/2005 [81]. Moreover, research conducted in Austria, assessing the degree of bacterial contamination of dairy products with *Escherichia coli* and *Staphylococcus aureus*, demonstrated that there was no difference in contamination levels between organic and conventional food [82]. Luukkonen et al. [74], in addition to milk quality, assessed the effects of the exclusion of nitrate and the introduction of a protective culture on the microbiological safety of organic Edam cheese and the counts of *Listeria* and non-pathogenic enterohaemorrhagic *Escherichia coli* (EHEC). Their study showed that nitrate failed to inhibit the growth of EHEC and *Listeria*. Thus, Edam cheese made from organic milk without added KNO_3_ posed no additional health risk with respect to *Listeria* and EHEC in comparison to Edam cheese from conventional milk. The use of a protective culture containing *Lactobacillus rhamnosus* LC705, however, inhibited the growth of *Listeria* in Edam cheeses from organic milk.

The total bacterial count in cheese depends mainly on the bacteria in raw milk, production conditions, starter culture activity, degree of acidification, maturation time and conditions, and treatment during ripening (in the case of ripened cheeses). The higher total bacterial count in ripened organic cheeses may be due to the shorter maturation time compared to cheese produced under industrial conditions. In the case of curd cheese and unripened acid rennet cheese, a higher TBC may result from excessively high temperature, improper storage temperature, and failure to maintain the cold chain during cheese distribution and sale.

Overall, there were no significant differences in the prevalence of bacteria in organic and conventional food products. Irrespective of the production system, dairy products are constantly exposed to microbiological health safety risks.

It should be emphasized that while research is carried out on the microbiological quality of dairy products, the results are often a cause for concern, and scientists choose not to publish them for fear that they will be rejected for publishing or raise controversy in the scientific community and the public. There is certainly much to be done to ensure the microbiological safety of food products, including perishable products such as milk and unpasteurized dairy products, in all parts of the world. Dairy farmers need to be educated about hygiene, especially on small family farms processing milk. High-quality raw milk is an important factor in obtaining high-quality products (which is crucial in cheese making) and affects their shelf life as well.

### 4.3. Physicochemical Quality

#### 4.3.1. Acidity

Acidity is a physical parameter indicating the freshness of raw milk. In an assessment of raw milk obtained from Simmental cows from organic farms, Wójcik-Saganek [57] obtained active acidity of 6.75 and potential acidity of 7.45 °SH, while the corresponding values from conventional farms were 6.73 and 7.29 °SH. The differences were not statistically confirmed.

According to *Codex Alimentarius* [83], titratable acidity, in addition to microbiological and chemical criteria, should be used to detect unacceptable conditions in milk products. Titratable acidity shows the buffering capacity of milk and indicates any changes in the concentration of acidic compounds in milk, even if the pH remains unchanged [84]. It should be noted that starter cultures are often used in dairy production (mainly for fermented beverages and some cheeses), and these essentially determine the product’s acidity. Wichrowska and Wojdyła [85] tested organic and conventional yoghurts available on the Polish market. The pH value, titratable acidity, and lactic acid content did not differ significantly between the two types of products. The active acidity of organic yoghurts was on average 4.42, potential acidity was 42.7 °SH, and lactic acid content was 0.96 g/100 g. The corresponding values for the conventional products were 4.50, 40.4 °SH, and 0.91 g/100 g. It is very common in conventional production to add powdered milk or milk protein to the product. This increases the lactose content in milk, which in the fermentation process decomposes into lactic acid, increasing the acidity of natural yoghurt. Results presented by Brodziak et al. [86] from their assessment of the acidity of organic pasteurized milk used for yoghurt production, expressed as active acidity (pH) and potential (titratable) acidity, in °SH, were consistent with Polish requirements for drinking milk [87]. According to these criteria, the active acidity (pH) of pasteurized milk should be in the range of 6.6–6.8, and titratable acidity should be 6.0–7.2 °SH. The acidity of the experimental yoghurts was also in compliance with the standard [88], according to which titratable acidity expressed as lactic acid content should not be lower than 0.6%. The lactic acid content in the organic yoghurts produced in that study varied depending on the production season and the type of starter culture.

#### 4.3.2. Proximate Chemical Composition

The content of basic components of milk, i.e., total protein, including casein, fat, and lactose, which together with minerals form the dry matter of milk, is important in processing. When purchasing raw milk, dairy plants prefer high content of protein, including casein. This is especially important in cheese making. In recent years, milk fat had not been as highly valued as it was in the past. However, significant increases in world butter prices have now led to renewed interest in milk fat, mainly in conventional production [89,90]. In April 2021, one of the leading dairies in Poland offered producers a litre of conventional milk for 1.5065 PLN as the starting price for 4.33% fat and 3.4% protein, with an additional rate of +0.060 PLN/L per unit of fat and +0.285 PLN/L per unit of casein [89].

Many studies have assessed the basic nutritional value of organic and conventional milk. According to Nauta et al. [59], Dutch farmers who converted to organic farming during the late 1990s represented a specific group of farmers distinct from conventional farmers. This was reflected in lower milk yields and lower milk fat and protein percentage after conversion compared to conventional farms. During conversion, significant changes occurred in milk production and in protein and fat content. Luukkonen [74] demonstrated that Finnish organic milk contained significantly less fat (4.17%, *p* ≤ 0.01) and protein (3.30%, *p* < 0.001) than conventional milk (4.24% and 3.38%, respectively), while organic milk contained significantly more lactose (4.79% vs. 4.74%, *p* ≤ 0.01). Partially similar results were obtained by Toledo et al. [91] from 31 organic dairy farms in Sweden for raw milk samples collected once a month for one year. The authors obtained similar results for fat, as they observed a significantly lower fat content in milk from large organic farms (40–60 animals) than in milk from large conventional farms. However, no differences were noted between the protein content of organic and conventional milk. A Polish study [61] assessed raw milk from Simmental cows from organic and conventional farms (traditional and intensive). The authors reported that milk obtained from organic and traditional farms was a significantly (*p* ≤ 0.01) poorer source of total protein (3.24% and 3.33%, respectively), including casein (2.47% and 2.53%, respectively), compared to the intensive system (PMR) (3.59% and 2.81%, respectively). Wójcik-Saganek [57] also found that the milk of Simmental cows kept on organic farms was a significantly (*p* ≤ 0.01) poorer source of total protein (3.15%), including casein (2.46%), compared to cows of this breed from conventional farms (by 13% and 5%, respectively). Therefore, it can be assumed that the production system in organic and traditional farms did not fully satisfy the cows’ requirements for nutrients. Other authors have also demonstrated significantly lower content of protein, including casein, in organic milk compared to conventional milk [4,59,71,92]. According to Zagorska and Ciprovic [92], the lower protein content in organic milk was caused by the lower amount of starch in the feed, which was linked to the smaller proportion of concentrated feeds. According to Kruczyńska [93], the use of maize silage in cow diets promoted bacterial protein synthesis in the rumen and had a positive effect on its quantity in milk. These relationships corresponded with the results of our own research, in which higher protein content was obtained in milk produced in the intensive system, where cow feeding was based primarily on maize silage. Vicini et al. [94] noted an increase in protein content in organic milk compared to conventional milk. In contrast, Luukkonen et al. [74] observed no effect of the organic and conventional production systems on milk protein content. Stergadis et al. [95] showed that the amount of protein, including casein, was significantly higher in organic milk than in conventional milk.

Fat is the main energy component of milk and dairy products. Its quantity is indicative of the quality of raw milk. It is one of the substances that has a direct impact on the preferences of consumers of dairy products. Fat is responsible for the palatability of milk and dairy products because volatile compounds responsible for flavour dissolve in it, giving the products more or less desirable attributes [71,96]. Król et al. [61] found that milk obtained from Simmental cows on organic farms had the lowest fat content (3.80%, *p* ≤ 0.01), compared to traditional (3.90%) and intensive systems (PMR) (4.10%). Together with the lowest total protein content, mentioned above, this also translated into significantly the lowest dry matter content (12.39%, *p* ≤ 0.01). Similar results were obtained by Wójcik-Saganek [57], who recorded statistically significantly (*p* ≤ 0.01) the highest fat (4.15%) and dry matter (13.07%) content in the raw milk of Polish Holstein-Friesian cows kept in an intensive system (TMR) compared to organic (about 10% and 7% less, respectively) and traditional systems (by approximately 3% and 2%, respectively). In contrast, Palupi et al. [97] reported higher total fat and protein contents for organic milk, while a meta-analysis by Średnicka-Tober et al. [62] found no significant difference in total fat and protein content between organic and conventional milk. Nahar et al. [98] reported that feeding cows green forage and concentrates increased dry matter content, including milk fat and protein. The feeding system exerts the greatest influence on the level of milk fat, and to a lesser extent determines the amount of protein. Changes in the level of fat and its composition largely depend on the concentration, composition, and form of crude fibre, as well as the starch and sucrose concentration in the diet. Total protein content in milk depends in part on the amount of available energy in the diet [99,100].

The content of basic milk constituents directly translates into the nutritional value of the final product. A meta-analysis by Palupi et al. [97], based on 13 articles (not all of which focused on basic nutritional value), showed that organic dairy products had significantly higher content of protein, with a medium cumulative effect (±95% confidence interval) of 0.56 ± 0.24, and fat (0.21 ± 0.18, respectively—small cumulative effect), than conventional products. Brodziak et al. [86] assessed the physicochemical properties of organic yoghurts in relation to milk treatment (raw milk obtained directly from organic farms, partially skimmed, and heat-treated in the laboratory vs. organic milk purchased at a shop and pasteurized at a dairy), production season, and starter culture. The yoghurts contained on average 10.96% dry matter, including 3.71% total protein.

### 4.4. Bioactive Compounds

#### 4.4.1. Whey Proteins

Whey proteins are a very important group of milk proteins, although they constitute only 20–25% of total protein (the remaining 75–80% is casein). Albumins, i.e., α-lactalbumin (α-LA), β-lactoglobulin (β-LG), and bovine serum albumin (BSA), make up approximately 75% of whey proteins—Table 3. They also include bacteriostatic substances, i.e., immunoglobulins, lactoferrin, lactoperoxidase, and lysozyme, which constitute 1–2% of total milk proteins. These proteins exert multi-faceted, positive effects on the human body, including antimicrobial (antiviral and antibacterial), anticancer, immunomodulatory, and antioxidant properties. They are an excellent source of energy, essential amino acids, and peptides [101,102,103,104].

Only a few studies have focused on evaluating the content of whey proteins in organic cow milk. Brodziak et al. [86], in a study conducted on the milk of Simmental cows kept in different production systems, reported that the production system did not affect the total content of whey proteins in milk (0.72 g/L in the traditional and organic system versus 0.70 g/L in the intensive system). It did, however, statistically significantly affect individual proteins, i.e., β-lactoglobulin (*p* = 0.001), α-LA (*p* = 0.071), BSA (*p* = 0.016), lactoferrin (*p* = 0.001), and lysozyme (*p* = 0.001). Milk from low-input farms (using organic and traditional systems) had a higher content of these whey proteins compared to intensive farms (PMR system). This was particularly true of proteins with antimicrobial properties, i.e., lactoferrin and lysozyme. Milk produced by cows raised in the intensive system was a poorer source of these proteins (by about 10%) than milk from the extensive systems (organic and traditional). The highest concentrations of lactoferrin (123.8 mg/L) and lysozyme (11.14 µg/L) were recorded in milk from certified organic farms. Similar relationships were obtained by Zagorska [105], who reported a nearly twofold higher concentration of lactoferrin in milk produced in the organic system compared to TMR. According to the author, the biologically active substances with immunomodulatory properties present in green fodder directly affected the level of lactoferrin and lysozyme in the raw milk. These dependencies were also confirmed by Wójcik-Saganek [57], who obtained 125.9 mg/L of lactoferrin in raw milk from organic production, 109.8 mg/L from conventional production, and 96.8 mg/L (23% difference) from intensive production (TMR). The corresponding values for lysozyme were 11.17 µg/L, 9.92 µg/L, and 6.90 µg/L (38% difference). On the other hand, Turner et al. [106] found that unrestricted access to pasture (ad libitum) did not increase the lactoferrin concentration in milk. This was confirmed by Kuczyńska et al. [51], who recorded higher lactoferrin content (0.387 g/L) in organic milk obtained in the summer on farms with limited access to pasture compared to farms using unrestricted green forage (0.240 g/L), although the difference was not confirmed statistically. The amount of lysozyme in that study was on average 16.04 µg/L on farms with unlimited access to pasture and 17.76 µg/L on farms with limited access.

Mackle et al. [107] noted a decrease in the content of primary whey proteins (albumins) in the milk of cows fed in an intensive system (with a limited amount of green fodder) compared to animals fed in traditional and organic systems (with additional concentrate feed). The authors found that increasing the amount of energy in the feed had a positive effect by increasing the whey protein concentration in milk. This was also demonstrated by Kuczyńska et al. [4], who obtained statistically significant differences (*p* < 0.01) in the amount of β-LG in milk from organic and traditional systems (4.12 vs. 2.68 g/L in the summer). According to Brodziak et al. [2], the β-LG concentration was comparable in raw milk produced in organic and traditional systems, i.e., 3.32 and 3.26 g/L, respectively. This was 0.10 g/L more than in the PMR system. Highly significant differences (*p* ≤ 0.01) in β-LG content were recorded by Król et al. [108], with a higher concentration (0.34 g/L) in the milk of grazing cows. Similar relationships were shown for α-LA by Brodziak et al. [109], with significantly (*p* ≤ 0.05) more of this protein in the milk of three local breeds of traditionally farmed cows (on average 1.17 g/L vs. 1.08 g/L in the intensive PMR system). This was also demonstrated in research by Wójcik-Saganek [57]. No statistically significant differences in milk α-LA content depending on the production system (organic vs. conventional farms) were reported by Kuczyńska et al. [4].

No studies by other authors have focused on evaluating whey proteins in organic dairy products. Brodziak et al. [2] determined the content of selected undenatured whey proteins in natural yoghurt made from the milk of Simmental cows on a certified organic farm. The products contained 0.72 g/L undenatured α-LA, 0.74 g/L β-LG, and 35.08 mg/L lactoferrin. Another study focused on yoghurts made from bulk milk obtained directly from organic farms and from shops [86]. With the exception of β-lactoglobulin, yoghurt produced in the spring and summer proved to be of greater nutritional value and had higher content of bioactive whey proteins, especially those made from milk obtained directly from farms.

The amount of whey proteins decreases when raw milk is heated, because they are partially denatured; this mainly applies to β-lactoglobulin and bovine serum albumin [110]. This was also demonstrated by Ruprichová et al. [111] in commercial yoghurts, in which β-lactoglobulin was the most thermally unstable protein. Sakkas et al. [110] reported the following order for heat sensitivity of whey proteins: α-lactalbumin < β-lactoglobulin < bovine serum albumin < immunoglobulins. Importantly, however, these changes did not affect the biological value of the product, because the proteins formed complexes with other substances and did not precipitate from the milk. The content of undenatured whey proteins is one of the chemical indicators used to assess the heat load of milk during processing [110,112].

#### 4.4.2. Fatty Acids

Milk fat is a composition of over 400 fatty acids (FA). These are mainly saturated fatty acids (SFA), causing the greatest concern among consumers, followed by unsaturated acids, i.e., monounsaturated fatty acids (MUFA) and polyunsaturated fatty acids (PUFA). However, owing to recent advances in science, trans fatty acids and saturated fatty acids in milk have been reported to have beneficial effects. Despite their unfavourable reputation, it is likely that they can prevent the development of civilization diseases such as diabetes, obesity, and hypertension [113,114,115].

The profile and amount of fatty acids in milk are determined by the feeding system [6,7,56]. Fresh herbs and grasses in the cow’s diet contain a much higher quantity of unsaturated fatty acids, while maize silage has a higher concentration of linoleic acid [116]. This translates directly into their quantity in raw milk and products made from it, which is partially illustrated in Table 4. The literature shows that the TMR feeding system significantly reduces the amount of fat and fatty acids in milk, as the diet lacks an adequate amount of dietary fibre, while its starch content is high [117,118].

Comparison of the fatty acid profile of organic and conventional (traditional and intensive systems) milk has shown significant differences in its composition in favour of organic milk [5,53,57,62,92,125,126]. Benbrook et al. [54,127] and Średnicka-Tober et al. [62] also showed that organic milk contained more polyunsaturated fatty acids (PUFAs), including omega-6 and omega-3, than conventional milk. Kuczyńska [51] reported a lower n-6 to n-3 ratio (by more than 50%) and a lower level of n-6 polyunsaturated fatty acids in raw organic milk, while the n-3 PUFA level was higher. Ellis et al. [128], Collomb et al. [129], Butler et al. [130], Adler et al. [131], and Benbrook et al. [54] also confirmed a more favourable ratio of n-6 to n-3 acids in milk from organic farms, as well as higher n-3 PUFA content. This was due to the availability of fresh pasture vegetation, including oilseed plants, such as flax [132]. In the human diet, n-3 long-chain fatty acids perform a number of beneficial physiological functions. For example, intake of eicosapentaenoic acid (EPA) and docosahexaenoic acid (DHA) in the population is insufficient. These deficiencies may have negative effects on brain function, which may increase the risk of Alzheimer’s disease in the elderly or the risk of cardiovascular or osteoarticular diseases [133,134,135]. Organic milk has been shown to be a more valuable source of EPA, DHA, and alpha-linolenic acid, even in winter [53]. The authors reported that the content of unsaturated fatty acids was statistically higher in raw milk from cows fed green fodder than from the TMR system. Raw milk from organic farms examined by Kuczyńska [51] was distinguished in particular by significantly higher content of cis-9, trans-11 CLA (1.9-fold higher at *p* ≤ 0.001), and α-linolenic acid (C18:3 n-3; 2.1-fold higher at *p* ≤ 0.001) compared to conventional farms. Kuhnt et al. [136] and Benbrook et al. [54] also showed higher CLA content in organic milk than in conventional milk. Consumption of milk and dairy products rich in CLA is associated with a beneficial effect on human health, such as improved brain function, reduced risk of civilization diseases, and lower levels of blood lipids. CLA also exhibits anti-carcinogenic, immunostimulatory, and weight-reducing properties [137,138,139].

From a technological perspective, changes in the quantity of fat and fatty acids in milk and fermented milk products have a significant impact on processing efficiency [140]. Breeding work is currently being conducted to modify the fatty acid profile of raw milk and thus of dairy products [141]. Organic milk and fermented dairy products are a valuable source of conjugated linoleic acid (CLA) [137]. Bergamo et al. [142] demonstrated that organic products with an increased level of CLA also had increased levels of natural antioxidants (α-tocopherol and β-carotene). According to Brodziak et al. [143], organic yoghurts have a better fatty acid profile than conventional products. They showed that the content of certain free fatty acids (myristic and palmitic acids) and groups of free fatty acids (short-chain free fatty acids—SCFFA, long-chain free fatty acids—LCFFA, polyunsaturated free fatty acids—PUFFA and all FFA) increased statistically significantly in the organic yoghurts during storage. There are also several reports concerning cheese. Bergamo et al. [142] noted significantly higher cis-9 trans-11 C18:2 (CLA), linolenic acid (LNA), and trans-11 C18:1 (TVA) concentrations in organic buffalo milk and mozzarella cheese. Similar results were obtained for heat-treated cow milk and dairy products, with all organic samples containing significantly higher levels of these compounds than conventional dairy foods. Butler et al. [130] and Fanti et al. [144] observed higher levels of CLA in pasteurized milk obtained at different times of the year, which ranged from 0.69 to 1.68 g in 100 g of fat in the organic product, and from 0.55 to 0.71 g in 100 g of fat in the non-organic product. Higher CLA values were also reported for butter [142] and fermented milk [145,146]. According to Fanti et al. [144], consumption of a 200 g serving of whole organic milk would provide about 41 to 101 mg CLA, compared to 33 to 43 mg of CLA from conventional milk. However, some authors have reported no significant difference between fatty acids in organic and non-organic milk [91,128].

#### 4.4.3. Vitamins

Cow milk and its products are considered a valuable source of vitamins in the human diet, such as retinol (vitamin A), vitamin D_3_ (cholecalciferol), tocopherol (vitamin E), vitamin K_2_ (menaquinone), and β-carotene (provitamin A) [2,4,5,147,148,149,150]. Vitamins are compounds with high biological activity that are essential for the growth and proper functioning of the body. They are involved in numerous vital processes, supporting metabolism and improving the activity of enzymes and catalysing proteins. Vitamin A, together with β-carotene (provitamin A), supports the differentiation, growth, and development of cells of the nervous and skeletal systems, vision, and foetal development. Vitamin D_3_ plays a key role in the metabolism of calcium and phosphorus, conditioning proper mineralization of bones and teeth. It also has immunomodulatory and anti-cancer properties. Vitamin E is one of the most powerful antioxidants and inhibitors of cell ageing [151,152]. The dairy industry is interested in a high content of vitamin E and β-carotene, as they can prevent spontaneous oxidation of milk and fatty acids. Their content in raw milk differs significantly depending on the production system, specifically on the type of feed [2,4,5], as shown in Table 5. Fresh pasture sward and silage from meadow vegetation, legume plants, and mixtures of legumes with grasses have higher content of vitamin E and provitamin A (β-carotene) than preserved fodder. Therefore, organic milk should be a more valuable source of vitamins, including fat-soluble vitamins, as well as carotenoids such as β-carotene, lutein, and zeaxanthin [149,153,154].

Numerous studies have compared vitamin content in organic and conventional milk (Table 5). However, definitive interpretation of the results is difficult. Many variables may contribute to differences in their content in raw milk, of which one of the most important is their availability in the feed [156]. Kuczyńska [51] found higher content of vitamins E and D_3_ and a lower content of β-carotene in cow milk from organic farms compared to conventional ones. The differences are most likely explained by the high content of these vitamins or provitamins in green forage, as well the exposure of animals to the sun, which promotes vitamin D_3_ synthesis [157]. The authors demonstrated that complete supplementation with synthetic vitamins could be used to improve the content of these health-promoting substances in milk from conventional farms (this is not possible in organic farming). In this case, their content in conventional milk may be higher than in milk from organic farms. This was confirmed by studies carried out in Sweden during the winter feeding period, which showed no differences in vitamin contents in milk produced in organic and conventional herds [158]. The highest concentrations of vitamins (mainly vitamin E and β-carotene) are found in fresh feed [4,159]. Brodziak et al. [2] reported a moderately strong relationship between the content of β-carotene and vitamin E in milk based on a correlation coefficient of r = 0.432 (*p* < 0.05). Vitamin losses can be observed during wilting, ensiling, or storage of fodder [4,150]. According to Kalač [150], if silage is poorly prepared, both α-tocopherol and β-carotene could be significantly degraded, which in turn would reduce the content of functional compounds. Havemose et al. [160] demonstrated that feeding cows grass silage significantly increased the number of antioxidants in milk compared to cows fed maize silage. According to Puppel et al. [5], supplementation of the basal diet with maize grain improved antioxidant capacity and the degree of antioxidant protection in milk. The degree of antioxidant protection and total antioxidant status were highest when pasture herbage was dominant in the feeding treatment. Leiber et al. [161] showed that cows that graze on a high-quality, diverse pasture produced milk containing 86% more vitamin E than cows fed preserved feeds with a high proportion of concentrates. According to Jensen and Nielsen [162], an increase in the natural content of α-tocopherol in feed from 500 to 1000 mg/cow per day increased its concentration in milk from 0.6 to 1.1 µg/mL—nearly twofold. On this basis, Mogensen et al. [154] posited that increasing the daily supply of α-tocopherol in the feed by 100 mg would increase its concentration in milk by 2.6 mg. Of course, this would only be possible in conventional production. Brodziak et al. [2], Kuczyńska et al. [4], Bergamo et al. [142], and Butler et al. [155] reported a naturally higher content of α-tocopherol and β-carotene in milk from organic farms compared to conventional milk, including milk from an intensive system. Similarly, Stergadis et al. [95] showed that the amount of lutein and zeaxanthin was significantly higher in organic milk than in conventional milk. However, recent meta-analyses have confirmed a higher level of α-tocopherol [62], but not of β-carotene [62,97], in organic milk. Comparable amounts of vitamin E and β-carotene in organic and conventional milk were recorded by Ellis et al. [163], but vitamin A content proved to be higher in milk from the traditional system. A similar relationship was found by Fall and Emanuelson [164], who compared raw milk obtained from organic and conventional dairy herds in winter. In comparison with the study by Ellis et al. [163], Chotyakul et al. [165] observed markedly lower concentrations of vitamins (vitamins A and E and β-carotene) in organic and conventional milk. Brodziak et al. [2], in a study on the Simmental breed, obtained the highest vitamin A levels in milk produced in systems based on pasture fodder (on average 0.468 mg/L in the organic system and 0.443 mg/L in the traditional one). Milk from intensive production (PMR system) contained almost 25% less of this vitamin (0.443 mg/L). Higher vitamin A content in the milk of cows fed a diet based on green forage was also described by Strusińska et al. [159].

Król et al. [3], Kuczyńska et al. [4], Puppel et al. [5], and Brodziak et al. [2,109] also analysed the content of vitamin D_3_ in milk. In animals spending time in pasture, ultraviolet (UV) rays from sunlight induce synthesis of vitamin D_3_ from 7-dehydrosterol present in the skin [2]. Thus, milk from cows that spend more time in pasture should be a more valuable source of this vitamin. This was confirmed by Brodziak et al. [2] and Kuczyńska et al. [100], particularly in the case of cows raised on certified organic farms, due to the specific nature of this type of production. Organically produced milk contained 0.768 µg/L of this vitamin, which was 0.038 µg/L more than in the traditional system and 0.144 µg/L more than in milk from the intensive system. Król et al. [3] recorded 0.653 µg/L of vitamin D_3_ in the milk of Simmental cows raised on intensive farms.

Vitamins in organic products have not been well researched. Bergamo et al. [142] obtained significantly (*p* < 0.05) higher α-tocopherol (vitamin E) concentrations in organic buffalo milk and mozzarella cheese than in conventional products. However, retinol (vitamin A) concentrations were lower in organic milk (*p* < 0.01) and cheese (*p* < 0.05). Similar results were obtained for heat-treated cow milk and dairy products, with significantly higher α-tocopherol and β-carotene concentrations in all organic samples than in conventional dairy foods. Brodziak et al. [86] tested natural yoghurts produced from organic milk obtained from an organic farm and from organic milk purchased in a shop. Yoghurts produced from bulk milk obtained from the farm were of greater nutritional value and had higher content of bioactive lipophilic vitamins than milk from the shop; the yoghurts contained 0.231 mg/L β-carotene (*p* ≤ 0.05), 0.408 mg/L vitamin A (*p* ≤ 0.01), 0.638 μg/L vitamin D_3_ (*p* ≤ 0.01), and 1.709 mg/L vitamin E (*p* ≤ 0.05). The effect of the production season also proved significant, in favour of the spring and summer season.

#### 4.4.4. Minerals

Milk and dairy products are good sources of minerals, such as calcium, potassium, zinc, selenium, phosphorus, and magnesium. Calcium is present in bones and teeth and is responsible for their mechanical strength. It is also involved in the control of heart contraction and supports nervous system functioning. Moreover, it reduces the permeability of blood vessel walls and has anti-inflammatory properties, alleviating skin and food allergies. Cu, Zn, Mn, and Fe play an important role in metabolic functions, such as maintaining bone health, regulating osmotic pressure, and involvement in muscle contractions. On the other hand, toxic elements such as lead, chromium, mercury, or cadmium may be present in milk and dairy products [166].

The most significant variation in the content of minerals in milk is determined by environmental factors, including the cow feeding system. The content of minerals in milk depends mainly on their concentration in fodder, and this in turn is associated with local soil and climatic conditions, as well as mineral and vitamin supplements in the cows’ diet [26]. Factors influencing the mineral composition of soil and pastures include fertilizers, the amount of sewage sludge generated, soil type, or the proximity of mining and industrial areas [125]. In conventional agriculture, soil fertility can be increased by using mineral fertilizers enriched with selected microelements. Cow diets are also supplemented with mineral mixtures. Both of these methods are restricted in organic farming. On-farm fodder is the main source of minerals on organic farms. Green forage from legume plants provides high amounts of calcium and magnesium, while cereal grains provide phosphorus, wheat bran is a source of magnesium, and green forage contains small amounts of sodium. In general, mineral supplementation via salt licks should be used to meet nutritional requirements [30,157,167].

The influence of pasture feeding on the mineral composition of milk is not clear. Kuczyńska [51], Wójcik-Saganek [57], Hermansen et al. [168], Gabryszuk et al. [169], and Koperska et al. [170] have demonstrated that organic milk is a poorer source of minerals than milk from conventional farms. The content of macro- and micronutrients in milk differed significantly statistically depending on the production system. Koperska et al. [170] reported that milk from organic farms had significantly (*p* ≤ 0.05) lower content of most of the analysed elements (Ca, Mg, Zn, Mn, and Cu). Litwińczuk et al. [26] observed the highest levels of minerals in milk from farms where the cows were fed traditionally (extensively). Milk from the organic system contained the lowest amounts of Ca, Na, Mg, Zn and Fe. The values for copper were comparable. The highest potassium content was found in organic milk, which could be due to the relatively large amount of this element in fodder from grasslands. Concentrations of iodine and selenium were significantly lower in organic milk than in conventional milk from cows fed concentrate feed [26]. This difference was even more pronounced in the summer, due to the increase in the proportion of green pasture forage in the diet [169,171,172]. Walther et al. [173] also showed a significantly lower concentration of iodine in organic milk than in conventional milk. This was true not only of the raw milk, but also the drinking milk produced from it. Organic UHT milk contained on average 36% less iodine than traditional UHT milk. A similar relationship was found by Flachowsky et al. [174], who reported that organic UHT milk contained 30–42% less iodine than conventional UHT milk. Pilarczyk et al. [175] found that organic milk from cows whose diet was rich in hay and maize silage had significantly higher selenium content than conventional milk. On the other hand, Fall and Emanuelson [164] reported nearly identical levels of Se in organic and conventional milk, which the authors argued was due to similar diets.

Table 6 compares the mineral contents of milk from different production practices. The raw milk from conventional production was a more valuable source of minerals (with the exception of potassium).

Zwierzchowksi and Ametaj [176] compared levels of heavy metals As, Ni, Al, Cd and Pb in organic and conventional milk. Conventional milk proved to be more contaminated (*p* < 0.05). The greatest differences were obtained for Al concentration, which was 6.5 times higher in conventional milk. 

The content of minerals in raw milk—mainly calcium, but to some extent phosphorus as well—affects the technological process. Unfortunately, the literature lacks comprehensive studies assessing mineral content in dairy products, particularly organic ones. Król et al. [147] found that the content of minerals in tvarog (traditional Polish acid curd cheese) did not depend on their content in the raw milk. According to Lucas et al. [177] and Manuelian et al. [178], differences in the mineral composition of cheese result from the degree of acidification of the raw milk, as well as from technological factors such as heating or salting.

### 4.5. Mycotoxins

Mycotoxins are compounds produced by moulds, especially of the genus *Aspergillus*, but also *Penicillium* and *Fusarium*. Mycotoxins commonly present in animal feed include aflatoxin (mainly B1), ochratoxin A, and zearalenone [179,180,181]. Aflatoxin B1 poses the greatest threat to human and animal health, as aflatoxin B1 and M1 are classified by the International Agency for Research on Cancer as carcinogenic and mutagenic compounds [182]. Mycotoxins also contribute to the development of numerous other diseases in animals and humans, including mycoses and mycotoxicosis [183].

Mycotoxins are widespread throughout the world, and their presence is determined by various factors (e.g., latitude, type of crop, agrotechnical procedures, and feed storage conditions). Currently, according to FAO estimates, about 25% of cereal grain in the world—and according to some sources up to 40%—is contaminated with at least one mycotoxin [9]. In silage, the percentage of samples infected with various types of moulds is much higher. In the case of deoxynivalenol (DON) and zearalenone (ZEN), two of the most prevalent mycotoxins in temperate climates, the percentages in grain samples are 60% (DON) or even 80% (ZEN) [181]. Animal feeds can become contaminated at any time—during plant development in the field, or during harvest, processing, storage, and transport. As feed components can pass into animal tissues and milk, feed should be of high quality, nutritional value, and microbiological purity. Often, however, meadow and pasture sward can be infected with moulds, and thus the green forage consumed by cows contains their metabolites. Silage may also be a source of mycotoxins, which may be present in contaminated raw material used for ensiling or produced by moulds developing during an improperly conducted ensiling process [183].

Cows whose feed is contaminated with aflatoxin B1 metabolize it in the liver to a less toxic metabolite, aflatoxin M1, which is secreted in the milk. The aflatoxin in milk has shown a tendency to increase during the winter feeding period [184]. Aflatoxin M1 is also detected in the milk of women that consume dairy products contaminated with this mycotoxin [185]. It should be noted that aflatoxin M1, in both raw milk and dairy products, is not broken down during heat treatment (pasteurization or UHT) or in further stages of production of dairy products, such as cheese, butter, or cream [186,187].

In order to protect animal health and the quality of raw milk, the maximum content of aflatoxin B1 has been specified as 0.02 mg/kg in feedstuffs for dairy cattle and 0.005 mg/kg in compound feeds [188]. Due to the adverse effects of aflatoxin B1 on the human body, the European Commission established the highest acceptable levels of aflatoxin M1—a metabolite of aflatoxin B1—in milk. According to Commission Regulation (EC) No 1881/2006 [189], its level may not exceed 0.050 µg/kg in raw milk and milk subjected to heat treatment, and 0.025 µg/kg in preparations for infants.

Regulatory levels and standards for mycotoxins vary in different parts of the world. At the global level, the Codex Alimentarius Commission in CXS 193-1995 establishes maximum levels (MLs) for AFs (sum of AFB1, AFB2, AFG1, and AFG2, and, separately, for AFM1 in milk) [190]. The Codex standard also defines sampling plans and performance criteria for analytical methods for determining mycotoxins. It should be added that despite the fact that the maximum levels in milk for other mycotoxins have not been established, ochratoxin A, aflatoxins G1, G2, B1, B2 and M2, fumonisin B1, cyclopiazonic acid, zearalenone and its metabolites, and deepoxy-deoxynivalenol have also been found in milk [191].

The literature contains many reports on the content of mycotoxins in milk and dairy products from different production systems. There are two theories regarding the presence of mycotoxins in milk. According to the first theory, organic milk and dairy products contain more mycotoxins than in the case of conventional production, while the other theory states the opposite. However, there is little evidence to support the first theory [192]. Organically raised livestock are fed greater proportions of hay, grass, and haylage, which reduces the opportunity for mycotoxin-contaminated feed to result in mycotoxin-contaminated milk. Wyss [193] reported that many studies have shown lower aflatoxin M1 levels in organic milk than in conventional milk, and therefore, the risk was no higher for organic milk and dairy products. Aflatoxin M1 in milk and dairy products were also the subject of an Italian study by Vallone et al. [194]. Higher aflatoxin M1 levels were found in milk from cows whose feed was based on maize, especially in the autumn/winter season. A lower level of these compounds was noted in milk—both organic and conventional—than in cheese. Organic milk and organic Crescenza cheese had lower levels of aflatoxin M1 because the milk was obtained from dairy cows raised in better health and hygienic conditions. Becker-Algeri et al. [180], based on available studies, the vast majority of which concerned conventional production, noted seasonal dependencies in the content of mycotoxins in milk. Storage of feedstuffs in unsuitable conditions during colder times of the year is conducive to the development of fungi. The authors observed very high variation in the occurrence of aflatoxin M1 in raw and treated milk, as well as in other dairy products (from 0—Greece and Brazil—to 100% of samples—Turkey, Serbia, Brazil, and Thailand). A study conducted on 188 organic products from Turkey (cheese, UHT milk, butter, and yoghurt) showed that they can pose a serious health threat, as 55% of samples tested positive for aflatoxin M1, and the acceptable limit was exceeded in 38% of samples. This was likely due to the fact that no synthetic antifungal agents are used in organic production [179]. Kos et al. [195], in a study conducted in Serbia, detected mycotoxins in all analysed samples of raw conventional and organic milk, but also in conventional pasteurized and UHT milk.

Research on the content of mycotoxins in milk and dairy products must be continued, due to the harmfulness of these compounds. Good agricultural practices and good feed storage practices are tools that can be used to significantly alleviate this problem in order to obtain high-quality raw milk.

### 4.6. Technological Quality

Assessment of raw milk as a material for processing most often takes into account the content and proportions of its individual components, mainly non-fat dry matter, including total protein, casein, and minerals. The proportion of total protein to fat often plays an important role, e.g., in cheese making. Other important indicators of the technological quality of milk are, rennet clotting time, heat stability, enzymatic coagulation capacity, and fat dispersion. These factors determine the effectiveness of basic technological treatments and the shelf life of dairy products.

Rennet clotting time is a parameter indicating the suitability of milk for cheese production. It is the time required to form a coagulum. According to many authors [196,197,198], the rate of curd formation and its firmness is primarily determined by milk composition, including casein content and the proportions of its individual fractions. Wójcik-Saganek [57] found that rennet coagulation time was significantly (*p* ≤ 0.01) shorter (2:44 min) in organic milk than in milk from a traditional system (4:42 min). Organic milk coagulated faster and was therefore more suitable for cheese production. These findings are consistent with the results obtained by Barłowska et al. [199] in conventional milk production. The authors indicated that green forage in the diet of cows reduced the rennet coagulation time. According to Devold et al. [200], the milk coagulation process significantly affects the quantity and proportions of protein fractions (casein and whey proteins), minerals (calcium and magnesium), and citrates. The content of these nutrients, however, is determined by how the cows are fed.

Heat stability is defined as the ability of milk, particularly proteins, to retain its colloidal properties when exposed to high temperatures. The heat stability of milk is a very important indicator of its suitability for processing at high temperatures, i.e., for the production of dairy products with extended shelf life, such as UHT milk and cream or milk protein concentrates [56,201]. In practice, heat stability is most often measured as the time required for the coagulation of milk heated at 140 °C. Milk acidity is one of the factors that directly determine its heat stability. Heat stability gradually increases over the pH range from 6.4 to 6.7. It begins to decrease significantly at a pH above 6.7, reaching a minimum at pH = 6.9, and subsequently begins to increase again at pH > 6.9 [202]. Raw organic milk analysed by Wójcik-Saganek [57] was significantly less stable (2:32 min at *p* ≤ 0.01) than milk from conventional farms (4:52 min).

The optimal protein-to-fat ratio plays an important role in cheese production, in addition to high content of protein, especially casein. This translates into higher cheese yield and to a better chemical composition, sensory attributes, and rheological properties. A protein-to-fat ratio from 0.70 to 1.15 results in optimal yield of cheese with favourable physicochemical parameters [203,204]. Król et al. [61] found that although milk from organic farms had the lowest protein content (3.24%, *p* ≤ 0.01), it had the highest protein-to-fat ratio (0.88), due to it low fat content (3.80%, *p* ≤ 0.01), compared to milk from conventional systems (traditional—0.86, intensive—0.87). Brodziak et al. [2] obtained the same protein-to-fat ratio in organic milk.

Undoubtedly, the observed changes in raw milk properties and following dairy processing were most likely influenced by the factors connected with the production system, i.e., herd size, dominant breed, feeding practices, housing, and milking system. This was also indicated by Akkerman et al. [205] and Priyashantha et al. [206]. Generally, the type of dairy farming system showed a significant effect on many of the investigated milk quality traits.

## 5. Conclusions

It should be emphasized that comparing organic and conventional systems is difficult due to many accompanying factors. Research assessing organic milk quality is much less extensive than in the case of milk from conventional production which may result from the diversified interest in this type of products, sometimes their limited availability but also from the controversial nature of organic production. The available reports indicate that raw milk from organic farms is more valuable, particularly in terms of the content of health-promoting compounds, such as vitamins, fatty acids, whey proteins, and minerals. This stems from the fact that in organic farming the animals are kept in pasture. However, the hygienic quality of the raw milk raises some concerns, as confirmed by our own observations. There is clearly a need for corrective GHP measures on farms with respect to both the acquisition and processing of raw milk on site. Even basic principles are not always obvious to some producers. In many parts of the world, research should be initiated or continued to assess the quality of both raw milk and the products obtained from it and thus provide a more complete picture of the current situation. It should also be noted that organic milk production, and to some extent traditional milk production, is in line with two strategies proposed under the European Green Deal—biodiversity protection and ‘from farm to table’.

## Figures and Tables

**Table 1:** Quality of milk for cheese making ([19,22,24], own unpublished work).

Characteristic	Suggested Values	Comment
Visual/sensory characteristics		
Appearance	-	Should be typical of milk (creamy white colour, homogeneous, no free fat or froth).
Smell	-	Should be typical of milk (no atypical odours and taints).
Biochemical/physical characteristics		
Active acidity (pH value)	6.5–6.8	
Freezing point (°C)	≤−0.520	
Protein content (g/100 g)	≥3.3	
Casein content (g/100 g)	≥2.50	Higher casein content is associated with higher cheese yield.
Non-protein nitrogen content (g/100 g total nitrogen)	<6	
κ-casein content (g/100 g total casein)	>15	
Fat content (g/100 g)	>3.5	Should remain relatively consistent to avoid large changes in liquid-to-solid fat ratio and rheology of fat phase in cheese.
Fee fatty acid content (mg/kg)	<3.5	Should be low to avoid rancid off-flavours.
Protein to fat ratio	>0.8	It proves the high suitability of milk for technological purposes.
Lactose content (g/100)	>4.5	
Somatic cell count (cells/mL)	≤100 × 10^3^	Recommended ‘gold standard’ is ≤50 × 10^3^ in 1 mL of milk.
Total bacterial count (colony forming units (cfu)/mL)	≤30 × 10^3^	
Plasmin (AMC units/mL) ^a^	<0.18	
Plasminogen (AMC units/mL) ^a^	<0.18	
Antibiotics	Not detectable	
Inhibitory substances (washing, disinfecting agents)	Not detectable	
Trichloromethane (µg/kg)	<2	
Processability characteristics		
Formagraph – curd firmness time (A30, mm)	20–40	
Rennet coagulating time –RCT (min)	11–18	
Rheometer (G′, Pa)	50 Pa at 31 °C in 60 min ^a^	
Syneresis	nd ^b^	Gel should undergo syneresis readily on cutting (may be measured empirically by centrifugation under defined conditions, or µg/kg).

^a^ AMC—aminomethyl cumarin. ^b^ nd—not defined.

**Table 2:** Quality of milk for the production of fermented milk beverages ([25], own unpublished work).

Characteristic	Suggested Values	Comment
Visual/sensory characteristics		
Appearance	-	Should be typical of milk (creamy white colour, homogeneous, no free fat or froth).
Smell	-	Should be free of atypical odours and taints.
Biochemical/physical characteristics		
Solids-not-fat (SNF) content (g/L)	~140 g/L for stirred fruit yoghurt	Bulk raw milk contains 85–90 g/L, so it must be raised by heating.
	higher SNF for the ‘luxury’ or Greek-style yoghurt	
Protein content (g/L)	40–50	Bulk raw milk contains ~33 g/L, so it must be raised. The higher the protein content in milk, the stronger the yoghurt gel.
Fat content (g/L)	10–12	Bulk raw milk contains ~30–35 g/L. The recommended level of 10–12 g/L gives yoghurt a smooth, satisfying ‘mouthfeel’.
Lactose content (g/L)	~45	Forms the bulk of the SNF (the balance is minerals)

**Table 3:** Content of whey proteins in cow milk from various production practices and in natural yoghurt (own work based on: [2,3,100,109], [86] *).

Content	Raw Milk			Natural Yoghurt *
	Organic System	Traditional System	Intensive System	Organic System
β-Lactoglobulin (g/L)	3.32–3.35	3.26–3.58	3.01–3.28	0.65–1.57
α-Lactalbumin (g/L)	1.07–1.19	1.05–1.21	0.98–1.14	0.75–0.77
Bovine serum albumin (g/L)	0.43	0.44	0.41–0.49	0.40–0.41
Lactoferrin (mg/L)	123.8–125.9	109.80–130.62	94.01–121.23	22.19–25.76
Lysozyme (µg/L)	11.14	9.92–10.71	6.90–12.13	3.15–3.39

* publication relating to the product.

**Table 4:** Content of fatty acids in cow milk from different production practices and in cheese (g/100 g of FA, %) (own work based on [5,57,119,120,121,122], [123] *, [124] *).

Fatty Acids (FA)	Raw Milk			Cheese *	
	Organic System	Traditional System	Intensive System	Traditional System (Pasture)	Intensive System (TMR)
Saturated fatty acids (SFA)	66.28	59.03–64.74	67.69–71.41	64.61–67.47	70.71–71.72
Monounsaturated fatty acids (MUFA)	26.11–34.07	30.33–32.16	21.87–28.15	28.22–31.71	25.58–27.13
Oleic acid (c9 C18:1)	nd	16.10–22.66	16.16–17.20	22.48–24.00	21.13–21.49
Wakcenic acid (t11 C18:1)	nd	1.18–7.00	0.80–2.00	0.83	0.46
Polyunsaturated fatty acids (PUFA)	3.85–5.36	3.69–5.32	1.65–3.77	3.68–4.31	3.71
Eicosapentaenoic acid, EPA (C20:5 n-3)	nd	0.08	0.05	nd	nd
Conjugated linoleic acid, CLA (cis9 trans11)	0.83–1.53	0.54–0.93	0.42–1.19	1.12–1.53	0.36–0.46
Linoleic acid, LA (C18:2 n-6)	nd	1.17–2.18	1.4–2.39	2.53	2.04
α-linolenic acid, ALA (C18:3 n-3)	nd	0.49–1.25	0.39–0.42	0.98–1.21	0.41–0.67
γ-linolenic acid, GLA (C18:3 n-6)	nd	0.13	0.12	0.18	0.13
Proportion 18:3n3: 18:3n6	nd	0.60–2.77	1.26	0.72	0.62

FA—fatty acid; nd—no data. * publications relating to the product.

**Table 5:** Content of selected vitamins in cow milk from various production practices and in natural yoghurts (own work based on [2,4,5,51,100,109,142,147,155], [86] *).

Content	Raw Milk			Natural Yoghurt *
	Organic System	Traditional System	Intensive System	Organic System
A (mg/L)	0.468–0.800	0.410–0.556	0.347–0.465	0.352–0.408
D_3_ (μg/L)	0.461–0.768	0.610–1.212	0.589–0.700	0.556–0.638
E (mg/L)	1.358–2.655	1.656–1.953	1.075–1.302	1.649–1.709
β-karoten (mg/L)	0.195–0.580	0.231–0.252	0.175–0.190	0.222–0.231

* publication relating to the product.

**Table 6:** Comparison of mineral contents (mg/L) in milk from different production practices [26,125,147,172,173].

Content	Raw Milk	
	Organic System	Conventional System
K	1896.92	1844.37
Ca	971.33	1404.70–1417.76
Na	366.59	476.35
Mg	86.21	113.87–118.50
Zn	2.86–3.96	2.96–4.39
Fe	0.32–0.67	0.34–0.47
Mn	0.023–0.047	0.022–0.139
Cu	0.023–0.084	0.038–0.161
I	0.013–0.283	0.071–6.540
Se	0.002–0.020	0.008–0.040
Co	0.001	0.001
Sr	0.166	0.202

## Data Availability

Not applicable.
